# Influence of retinoic acid on mesenchymal stem cell differentiation in amyloid hydrogels

**DOI:** 10.1016/j.dib.2015.11.015

**Published:** 2015-11-17

**Authors:** Reeba Susan Jacob, Subhadeep Das, Dhiman Ghosh, Samir K. Maji

**Affiliations:** a Department of Biosciences and Bioengineering, Indian Institute of Technology Bombay, Mumbai 400076, Maharashtra, India; b IITB Monash Research Academy, IIT Bombay, Mumbai, India

**Keywords:** Amyloid hydrogel, Nanofibrils, Self-assembly, Stem cell, Tissue engineering, retinoic acid

## Abstract

This paper presents data related to the research article “Self healing hydrogels composed of amyloid nano fibrils for cell culture and stem cell differentiation” [1]. Here we probed the collective influence of all-trans retinoic acid (RA) and substrate properties (amyloid hydrogel) on human mesenchymal stem cell (hMSC) differentiation. Stem cells were cultured on soft amyloid hydrogels [1], [2] in the presence and absence of matrix encapsulated RA. The cell morphology was imaged and assessed via quantification of circularity. Further immunostaining and quantitative real time PCR was used to quantify various markers of differentiation in the neuronal lineage.

**Specifications Table**

**Table:** 

Subject area	*Biology, Material Science, Stem cell*
More specific subject area	*Biomaterial, Stem cell differentiation*
Type of data	*Image (microscopy), graph, figure*
How data was acquired	*Phase contrast microscopy, Fluorescence Microscope, SEM etc.*
Data format	*Analyzed*
Experimental factors	*Amyloid hydrogel, retinoic acid*
Experimental features	*Human mesenchymal stem cells cultured on amyloid hydrogel loaded with retinoic acid.*
Data source location	*IIT Bombay, Mumbai, India.*
Data accessibility	*Data is provided in the article*

**Value of the data**

• Data is useful to have a better understanding of the influence of retinoic acid on mesenchymal stem cell differentiation seeded on amyloid hydrogels.
• Combined effect of RA and substrate stiffness on expression of neuronal markers in differentiating hMSCs.

## 1 Data

**Fig. 1:**
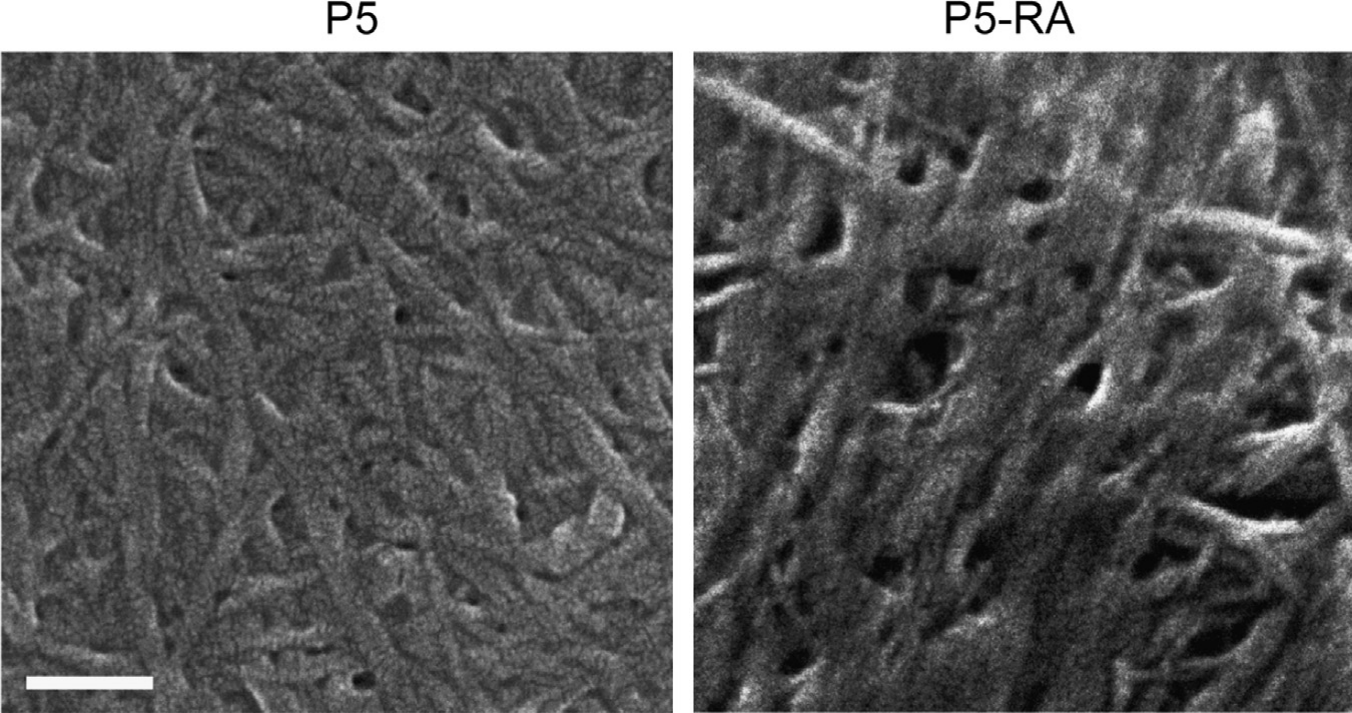
FEG SEM image of nano-fiber arrangement in P5 gel and P5 gel mixed with 10 μM RA. Scale bar is 100 nm.

**Fig. 2:**
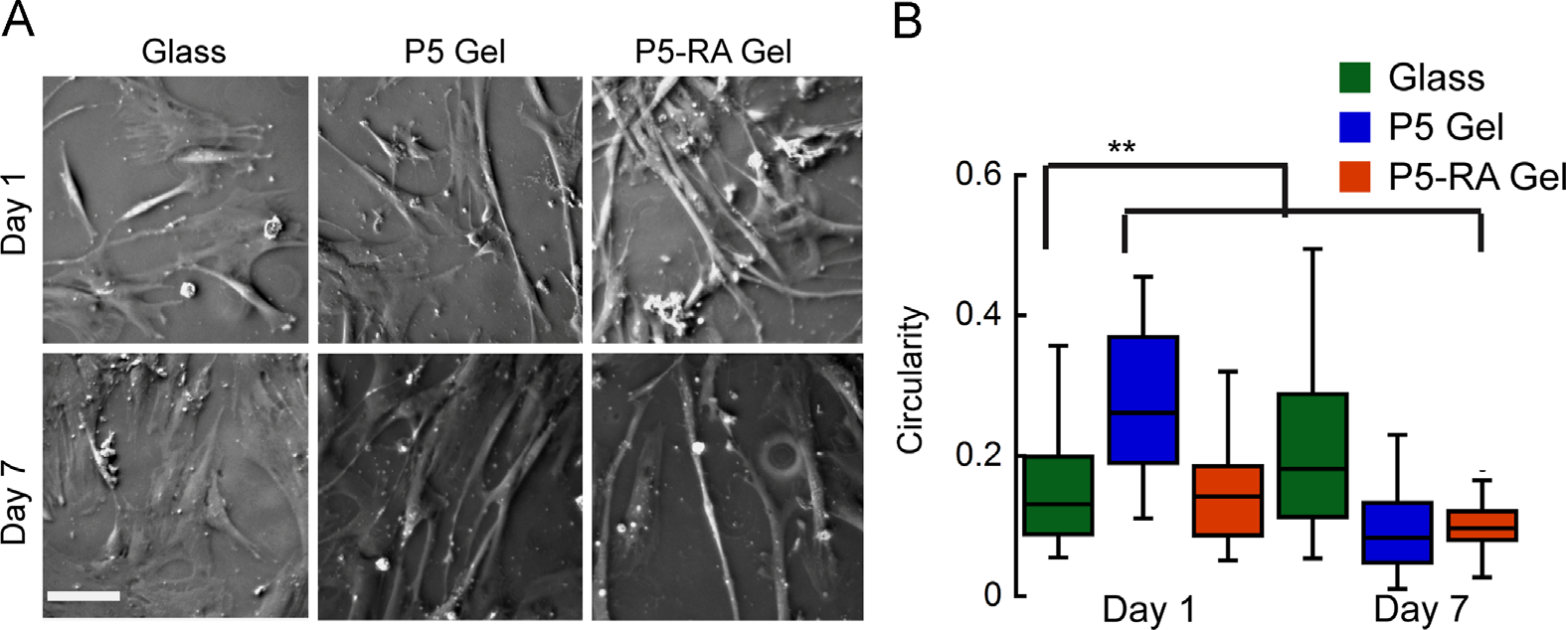
Influence of retinoic acid (RA) on hMSC differentiation. (A) Phase contrast images of hMSCs on glass, P5 gel and P5-RA gel surface after 1 day and 7 days of culture. Scale bar is 100 μm. (B) Circularity of hMSCs cultured on glass, P5 and P5-RA gels. The data are from 3 replicates. ** indicates statistical significance (*P*<0.001).

**Fig. 3:**
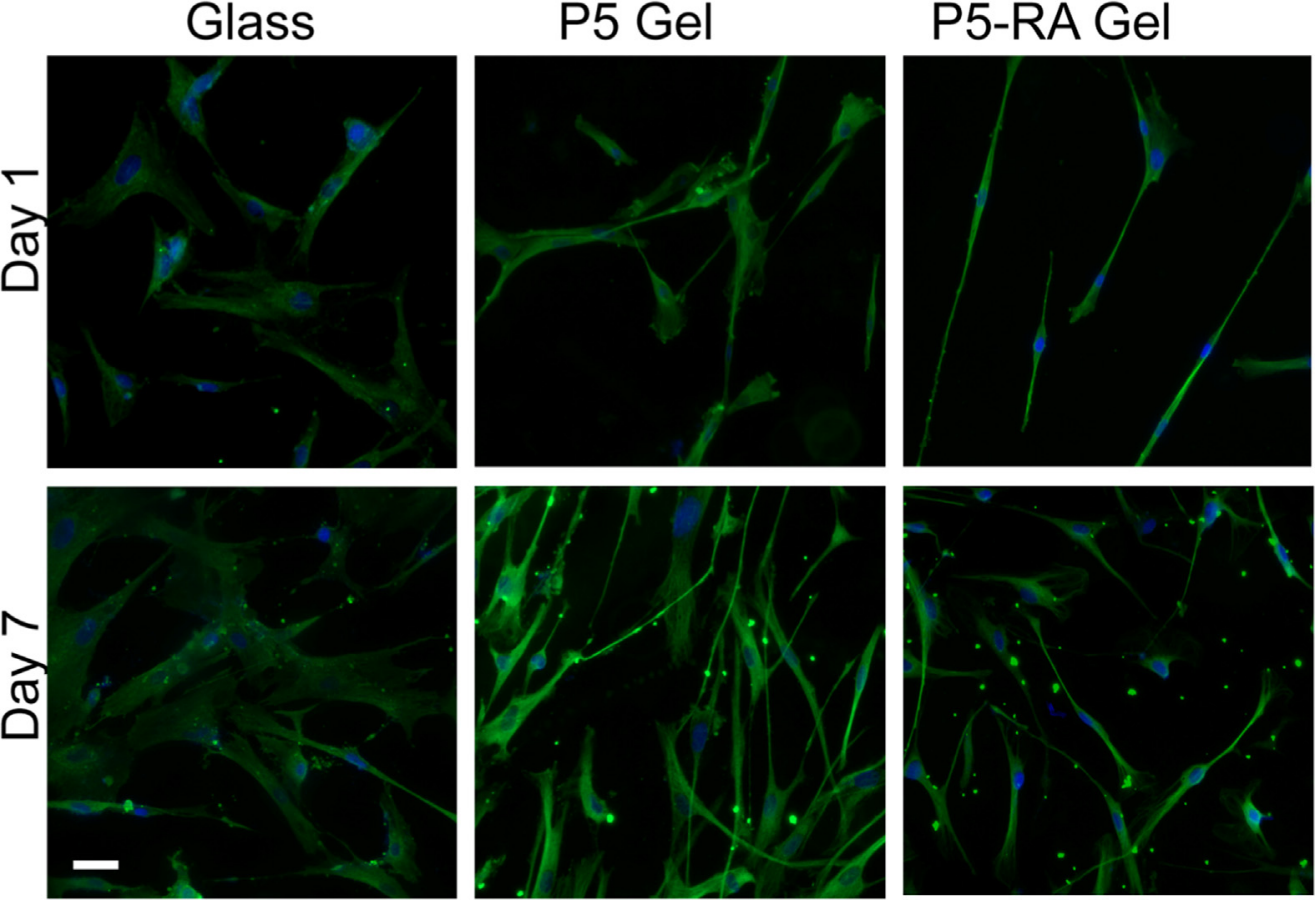
Immunocytochemistry of hMSCs. The hMSCs were grown on P5 and P5-RA gel for 7 days and was stained with neuron specific marker βIII tubulin (green) in cells. Nucleus is stained with DAPI (blue). Scale bar is 50 μm.

**Fig. 4:**
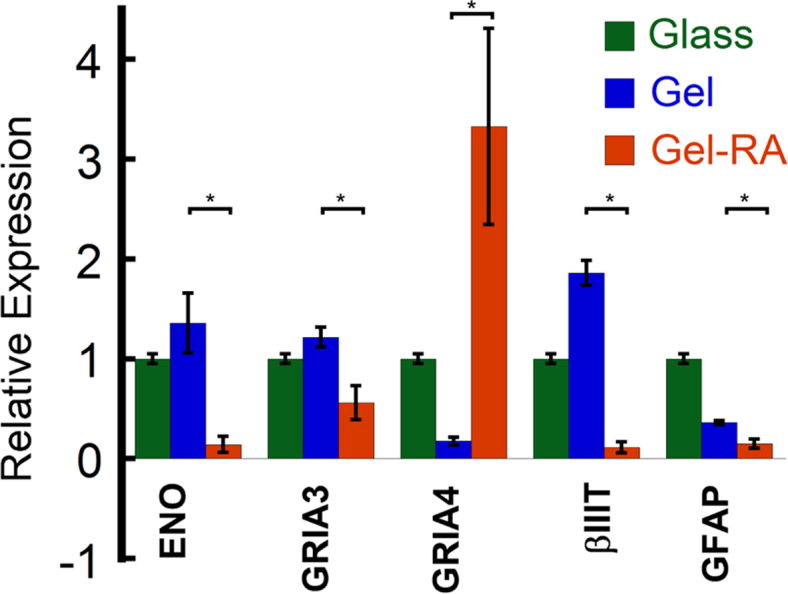
Gene expression profile of hMSCs after 7 days of culturing on gels and glass. On P5 gels, neuronal markers ENO and βIII Tubulin (βIIIT) were upregulated and astrocyte marker GFAP was down regulated. On P5-RA gels, only the glutamate ion-channel marker GRIA-4 was shown to be upregulated. Statistical analysis of gene expression was done between cells cultured on P5 and P5-RA gels with one way ANOVA; *P<0.05.

In addition to mechanical cues, stem cells are also sensitive to soluble cues and growth factors released in the extracellular space [3], [4], [5]. All trans retinoic acid (RA) is known to be a potent inducer of neuronal differentiation in neuroblastoma cell lines [6]. To probe the collective influence of RA and substrate properties on hMSC differentiation, 10 μM RA was mixed with the peptide solution during gelation. The morphology of RA entrapped gels (P5-RA gels) was studied via SEM (Fig. 1). We also cultured hMSCs on RA encapsulated P5 (Fmoc-VIV) [1] gels to observe their morphological differences with those cultured on glass and P5 gel alone (Fig. 2). Immunostaining (Fig. 3) and quantitative real time PCR (Fig. 4) was performed to ascertain the state of differentiation in the cultured stem cells.

## 2 Experimental design, materials and methods

### 2.1 Field-emission gun-scanning electron microscopy (FEG-SEM)

For characterizing the fibrillar morphology of amyloid gel encapsulated with RA, the P5 hydrogel (6 mg/mL) was briefly vortexed and mixed with 10 µM RA. This was casted on the stub and allowed to form gel, which was subsequently dried under vacuum overnight. The dried gels were sputter coated with platinum for 45 s at 10 kV voltages and 10 mA current. The gels were then imaged using JEOL Scanning Microscope-JSM-6700F. P5 gel without RA was used as control.

### 2.2 hMSc culture on RA gel

For studying the stem cell fate on the gel that was entrapped with RA, 100 μL of P5 peptide sol (obtained by vortexing the P5 gel of 6 mg/mL concentration) was mixed with 0.1 μL of 10 mM RA such that the final concentration of RA in gel became 10 μM. The RA mixed gel solution was then cast on a treated coverslip. hMSCs of cell density 1×10^4^ were seeded onto the surfaces of these gels and cultured. The hMSCs were imaged on day 1 and day 7 and the morphology of these cells were quantified in terms of circularity using image J (NIH, Version 1.47) wherein at least 50 cells were analyzed for each condition. Circularity is defined as (perimeter squared)/(4*pi*area), with 1 indicating a perfect circle [7].

Immunostaining was performed according to protocols described in our earlier publication [1].

### 2.3 Quantitative real time PCR (qPCR)

Quantitative real time PCR was performed with cell lysates derived from hMSCs cultured on P5 hydrogel loaded with RA. As a control only hydrogel P5 and glass was used. Cells were trypsinised, lysed and RNA was collected by TriZol (Invitrogen, USA) method using manufacturer’s protocol. The characteristic genes for neuronal differentiation were checked with pre-designed SYBR green primers from Sigma-Aldrich using SYBR green chemistry.

### 2.4 Statistical analysis

The statistical significance was determined by one-way ANOVA followed by Newman–Keuls Multiple Comparison post hoc test.

## Conflict of interest

The authors declare no conflict of interest.

## References

[1] Jacob R.S., Ghosh D., Singh P.K., Basu S.K., Jha N.N., Das S. (2015). Self healing hydrogels composed of amyloid nano fibrils for cell culture and stem cell differentiation. Biomaterials..

[2] Levental I., Georges P.C., Janmey P.A. (2007). Soft biological materials and their impact on cell function. Soft Matter.

[3] Discher D.E., Mooney D.J., Zandstra P.W. (2009). Growth factors, matrices, and forces combine and control stem cells. Science.

[4] Benoit D.S., Schwartz M.P., Durney A.R., Anseth K.S. (2008). Small functional groups for controlled differentiation of hydrogel-encapsulated human mesenchymal stem cells. Nat. Mater..

[5] Engler A.J., Sen S., Sweeney H.L., Discher D.E. (2006). Matrix elasticity directs stem cell lineage specification. Cell.

[6] Påhlman S., Ruusala A.-I., Abrahamsson L., Mattsson M.E.K., Esscher T. (1984). Retinoic acid-induced differentiation of cultured human neuroblastoma cells: a comparison with phorbolester-induced differentiation. Cell Differ..

[7] Thurston G., Jaggi B., Palcic B. (1988). Measurement of cell motility and morphology with an automated microscope system. Cytometry.

